# Lower serum 25-hydroxyvitamin D levels predict higher risk of DSPN in type 2 diabetes, and exhibit a non-linear association with the severity of DSPN

**DOI:** 10.3389/fendo.2026.1890018

**Published:** 2026-07-13

**Authors:** Tao Chen, Yi Feng

**Affiliations:** Department of Endocrinology and Metabolism, Longyan First Affiliated Hospital of Fujian Medical University, Longyan, China

**Keywords:** distal symmetric polyneuropathy, liquid chromatography – tandem mass spectrometry, propensity score matching, serum 25-hydroxyvitamin D, T2DM

## Abstract

**Background:**

Prior investigations have suggested a relationship in which vitamin D may be linked to distal symmetric polyneuropathy (DSPN), but these results are inconsistent. Moreover, few studies have examined the linear relationship between vitamin D levels and the severity of DSPN and no definitive clinical threshold has been identified. This study therefore investigated the connection between the level of serum 25-hydroxyvitamin D [25(OH)D] and the risk and severity of DSPN with special regard to the establishment of a clinically significant cutoff point to classify the risks.

**Methods:**

This multicenter cross-sectional study included 569 adults with type 2 diabetes mellitus (T2DM) from four hospitals in Fujian Province, China, between June 2022 and May 2023. Serum 25(OH)D_2_, 25(OH)D_3_, and total 25(OH)D levels were measured by liquid chromatography-tandem mass spectrometry (LC-MS/MS). DSPN and its severity were assessed by using a standardized five-item neurological examination and the Michigan Neuropathy Screening Instrument (MNSI). Multivariable logistic regression was applied to examine the relationship between 25(OH)D and DSPN. Propensity score matching (PSM) was performed to minimize baseline imbalances and validate the robustness of the findings. Restricted cubic splines (RCS) were employed to construct a dose-response curve and identify a possible threshold.

**Results:**

Compared with non-DSPN individuals (n = 148), those with DSPN (n = 421) had significantly lower concentrations of 25(OH)D (23.95 ± 5.14 vs. 32.31 ± 5.97 ng/mL) and 25(OH)D_3_ (22.88 ± 5.11 vs. 31.19 ± 5.70 ng/mL) (P < 0.001). By contrast, 25(OH)D_2_ concentrations presented no evident intergroup difference (P = 0.645). Quartile analysis demonstrated a significant downward trend in the risk of DSPN with increasing 25(OH)D levels (P for trend < 0.001). After controlling for potential confounders, 25(OH)D continued to exert an independent protective effect, and this conclusion remained valid even after propensity score matching. RCS analysis revealed that 25(OH)D and DSPN risk had a linear negative relationship, and a nonlinear relationship was observed with the severity of neuropathy with an turning point at about 26.1 ng/mL. Additional examination revealed that the severity of DSPN increased significantly as the level of 25(OH)D dropped below this point.

**Conclusion:**

25(OH)D was inversely associated with the risk of DSPN, and exhibited a nonlinear relationship with neuropathy severity. Notably, when 25(OH)D levels dropped below 26.1 ng/mL, the severity of DSPN appeared to accelerate markedly. Therefore, 25(OH)D levels have the potential to be a readily accessible and clinically useful indicator to screen and categorize DSPN risk in the early stages.

## 1 Introduction

Over recent decades, the public health burden posed by type 2 diabetes mellitus (T2DM) has escalated globally, cementing its role as a key driver of the growing prevalence of metabolic disorders worldwide. Its prevalence has increased significantly in the last 30 years, becoming a huge burden to the public health systems (1). The International Diabetes Federation highlights that the adult diabetic patients worldwide keep rising, with a nearly 45% increase projected by 2050 compared with the 589 million cases recorded in 2024 (2). Chronic and progressive complications caused by diabetes can progressively worsen patients’ health status and further increase healthcare costs (3). Among various chronic comorbidities of T2DM, diabetic peripheral neuropathy (DPN) accounts for the highest proportion, impacting over half of patients, with reported prevalence reaching up to 75% in some studies (4–7).

One of the most frequent forms of DPN, and amenable to early detection and treatment, is distal symmetric polyneuropathy (DSPN). As a length-dependent peripheral nerve disorder, it mainly causes symmetrical bilateral sensory impairments of the lower limbs, manifesting as numbness, tingling sensation, burning pain and typical stocking-pattern sensory disturbances (8). Upon the onset of DSPN, small nerve fibers are the main target. This kind of damage cannot be easily identified using traditional nerve conduction studies. A small-fiber injury occurs at the early stages of neuropathy, with a 2019 study published in The Lancet reporting that electrophysiological tests commonly measure large fibers. This can be used to justify the fact that traditional nerve conduction studies are less sensitive when used in the diagnosis of early-stage disease (9). Moreover, it is possible that small-fiber damage is at least partially reversible at this point (5), which underscores the need for early screening and prompt management to limit or prevent progression.

Nevertheless, past reports point out that a significant percentage of patients with DSPN can be asymptomatic over an extended duration, and thus they are usually underdiagnosed (10). The neuropathy can often be irreversible by the time clinical symptoms become apparent. Advanced DSPN may trigger serious adverse complications, including diabetic foot ulcers and lower-limb amputation, which impose a substantial physical and economic burden on patients and greatly consume public healthcare resources (3). Statistics indicate that up to 27% of direct medical expenditures for diabetes in the United States are attributable to DSPN (11). Compared with such societal costs, the personal burdens borne by patients are far greater: they endure persistent symptoms such as numbness and pain, and may even develop anxiety and depression, leading to a marked decline in quality of life (12). Such clinical characteristics strongly support the urgent requirement for optimized and standardized early screening protocols for DSPN. Current guidelines from diabetes associations in China, Canada, and the United States recommend a standardized five-item neurological examination for DSPN screening (8), which can be further combined with the Michigan Neuropathy Screening Instrument (MNSI) to evaluate disease severity (13).

The etiology and pathogenesis of DSPN remain unclear. Essentially, according to the latest 2023 findings, DSPN can be regarded as a state of metabolic impairment and bioenergetic failure of the peripheral nervous system (14). However, it is currently believed to be associated with genetic and environmental factors, inflammation, oxidative stress, microcirculatory ischemia and hypoxia, a lack of nerve growth factor, autoimmune processes, and vitamin deficiencies. Clinical evidence suggests that age, diabetes mellitus (DM) duration, lifestyle patterns, and metabolic disturbances, including hyperglycemia, hypertension, and dyslipidemia, may contribute to its development (13, 15–18). Nowadays, more and more diabetes researchers are interested in vitamin D. As a regulator of calcium-phosphate equilibrium and bone homeostasis, it is widely distributed in the body via vitamin D receptors and thus possesses numerous pleiotropic functions. These recently discovered extraskeletal functions include suppressing autoimmunity, reducing inflammatory responses and improving glucose metabolism. Evidence from a meta-analysis including 26 global studies with 6,277 T2DM patients indicated that reduced vitamin D levels are frequently observed in individuals with DSPN, and insufficiency may be associated with an elevated risk of this condition (19). Nevertheless, findings across studies have not been consistent. For example, Mendelian randomization studies have not provided evidence of causality (20).

Accordingly, this study used multicenter observational data to systematically examine the relationship between 25(OH)D and the incidence and severity of DSPN in T2DM patients. Through clarification of these associations, we sought to generate more clinical evidence to support the promotion of early screening and risk stratification of DSPN in normal clinical care.

## 2 Methods

### 2.1 Data source and study design

Participants were recruited from four hospitals located in Longyan, namely The First Affiliated Longyan Hospital of Fujian Medical University, the Longyan Hospital of Traditional Chinese Medicine affiliated with Xiamen University, Longyan Boai Hospital, and Shanghang County Hospital (Longyan). Prior to enrollment, written informed consent was secured from every participant. The minimum number of participants was calculated with G*Power software (version 3.1.9.4, Germany). With alpha set to 0.05 and statistical power maintained at 90%, a total of 180 cases were determined as the minimal sample requirement. In an effort to achieve a more representative sample while minimizing selection bias, the study ultimately included approximately 540 participants.

Eligible participants were 18–79 years old and had a confirmed diagnosis of T2DM based on the Chinese Guidelines for the Prevention and Treatment of Type 2 Diabetes (2020 Edition) (21). 1) Participants were deemed ineligible if any of the following criteria were present: diabetes types other than T2DM, including type 1 diabetes (T1DM), specific types of diabetes, and gestational diabetes; 2) acute unstable metabolic conditions, including diabetic ketoacidosis, hyperosmolar hyperglycemic state, and severe hypoglycemia; 3) acute inflammatory or stress conditions, or malignancy; 4) disorders affecting vitamin D metabolism, including thyroid or parathyroid diseases, osteoporosis, or related medications; 5) any vitamin D supplement use within the last month; 6) pregnancy or lactation; 7) history of myocardial infarction, angina, stroke, or other peripheral vascular diseases; 8) chronic alcohol abuse; 9) severe hepatic or renal dysfunction, defined as bilirubin >1.5 × ULN, alanine aminotransferase >2 × ULN, or eGFR <20 mL/min/1.73 m²; 10) psychiatric disorders, including schizophrenia or depression; 11) neuropathy due to other causes, such as cervical or lumbar spine disorders, cerebral infarction, Guillain-Barré syndrome, severe vascular disorders, drug-induced neurotoxicity, or uremic neuropathy.

### 2.2 DSPN diagnosis and severity evaluation

The diagnostic approach for DSPN followed the 2021 Expert Consensus on the Diagnosis and Management of Diabetic Neuropathy (22). A diagnosis required both a documented history of diabetes and evidence of neuropathy emerging either at the time of diabetes onset or during its subsequent course. In patients presenting with symptoms such as paresthesia, pain, or numbness, the presence of at least one abnormal finding among five neurological assessments (vibration, pressure, ankle reflex, temperature, and pinprick sensation) was considered sufficient (23). For asymptomatic individuals, at least two abnormal findings were required. Nerve conduction studies were further performed when the diagnosis remained uncertain.

The severity of the disease was also assessed according to the MNSI that was comprised of a patient-reported questionnaire (MNSI-Q), and a structured clinical examination (MNSI-PE). The questionnaire has 15 questions on the symptoms (two of them are not counted) with a maximum score of 13; the higher the score, the more severe the symptoms. MNSI-PE was an objective assessment of the foot condition (deformities, calluses, ulcer, etc.), ankle reflexes and vibration sensation.

### 2.3 Clinical data collection

A case report form (CRF) was designed based on the study protocol. Structured interviews were used to collect data, including age, gender, DM duration, smoking and drinking history, sun exposure, use of protective clothing or sunscreen, and skin tone. Anthropometric parameters — such as height, weight, waist circumference, and blood pressure — were also recorded.

### 2.4 Laboratory measurements

Morning fasting venous blood samples were collected by trained clinical staff after an 8–10 hour overnight fast. The analyzed biochemical parameters included glycated hemoglobin (HbA1c), fasting plasma glucose, triglycerides (TG), total cholesterol (TC), high-density lipoprotein cholesterol, low-density lipoprotein cholesterol, creatinine (Cr), and serum calcium levels. Insulin resistance (IR) was quantified using the HOMA2 software (version 2.2; University of Oxford Diabetes Centre, UK) (24). Using the Chronic Kidney Disease Epidemiology Collaboration (CKD-EPI) equation recommended in Kidney Disease: Improving Global Outcomes (KDIGO) guidelines, eGFR was estimated from serum creatinine together with age and ethnicity (25).

Serum 25(OH)D concentrations were quantified using liquid chromatography-tandem mass spectrometry (LC-MS/MS).

### 2.5 Statistical analysis

All case report forms were double-entered and processed with the help of EpiData 3.0. Statistical tests were done with R software (version 4.1.2). Categorical variables were displayed in the form of percentages and compared with the help of chi-square test. Continuous variables underwent normality testing; those following a normal distribution were presented as mean ± SD, while non-normally distributed variables were reported as median (IQR). Subjects were divided into 25(OH)D quartiles, with trend tests based on quartile medians. Logistic regression identified independent factors for DSPN, with forest plots summarizing univariable and multivariable results. 1:1 nearest-neighbor propensity score matching (PSM) was performed to match the characteristics of the baseline with a caliper width of 0.1. Restricted cubic splines (RCS) models assessed nonlinear associations of 25(OH)D with DSPN risk and MNSI-PE scores. Graphs were generated in GraphPad Prism 8. Statistical significance was defined as a two-sided P value < 0.05.

## 3 Results

### 3.1 Baseline characteristics according to DSPN status

**Figure 1:**
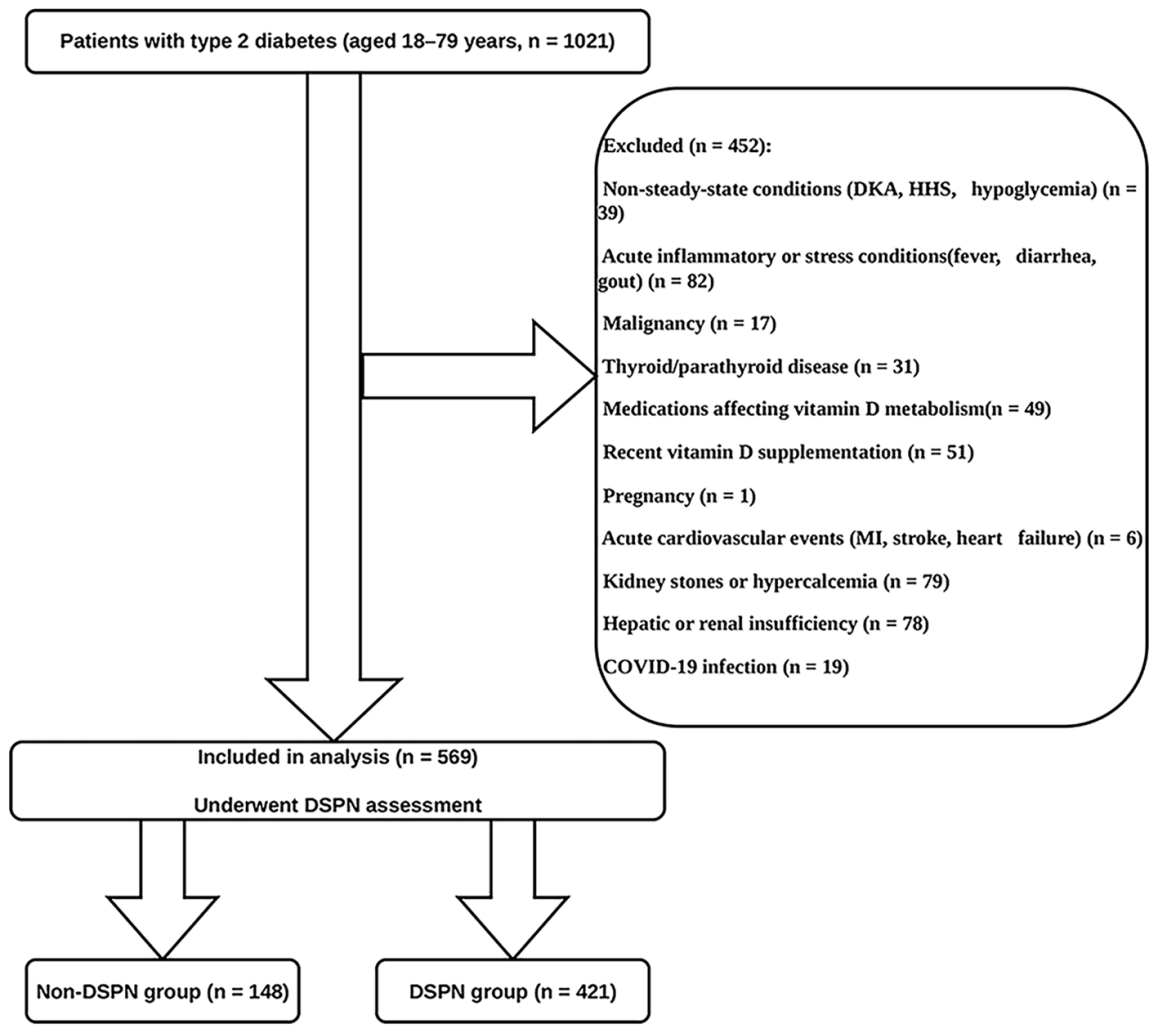
Flowchart of participant inclusion and exclusion.

The sample of this study was filtered among 1021 individuals aged 18–79 years who had established T2DM. After excluding ineligible subjects, a total of 569 people remained for analysis. According to DSPN status, participants were stratified into non-DSPN (n = 148) and DSPN (n = 421) groups (**Figure 1**). As shown in **Table 1**, patients in the DSPN subgroup were older, had a longer duration of diabetes, and exhibited higher IR levels, while they had a relatively smaller waist circumference compared to those in the non-DSPN subgroup (*P* < 0.01). However, no significant differences were observed in gender composition, smoking, or drinking (*P* > 0.05). Similarly, sun exposure levels and relevant environmental factors were also comparable between the groups (*P* > 0.05)(**Supplementary Table 1**).

**Table 1:** Baseline characteristics of the study participants.

Variable	Total (n = 569)	Non-DSPN (n = 148)	DSPN (n = 421)	Statistic	*P*
Age, years	58.36 ± 11.38	55.87 ± 11.44	59.24 ± 11.24	t = -3.117	0.002
Gender, n (%)				χ² = 3.270	0.071
Male	353 (62.04)	101 (68.24)	252 (59.86)		
Female	216 (37.96)	47 (31.76)	169 (40.14)		
Smoke, n (%)				χ² = 0.025	0.875
No	424 (74.52)	111 (75.00)	313 (74.35)		
Yes	145 (25.48)	37 (25.00)	108 (25.65)		
Drinking, n (%)				χ² = 2.458	0.117
No	398 (69.95)	96 (64.86)	302 (71.73)		
Yes	171 (30.05)	52 (35.14)	119 (28.27)		
DM duration, years	7.00 (2.00–10.17)	5.00 (1.00–10.00)	8.00 (3.00–11.00)	Z = -3.074	0.002
SBP, mmHg	129.35 ± 17.39	127.05 ± 16.46	130.16 ± 17.66	t = -1.871	0.062
DBP, mmHg	81.06 ± 10.96	80.94 ± 10.40	81.10 ± 11.17	t = -0.155	0.877
BMI, kg/m²	24.13 ± 3.36	24.51 ± 3.41	24.00 ± 3.34	t = 1.585	0.114
WC, cm	89.00 ± 11.73	91.34 ± 11.65	88.17 ± 11.65	t = 2.845	0.005
ALT, U/L	27.16 ± 19.58	28.39 ± 23.16	26.72 ± 18.16	t = 0.891	0.373
Ca, mmol/L	2.37 ± 0.16	2.38 ± 0.14	2.37 ± 0.16	t = 1.162	0.246
Cr, μmol/L	76.77 ± 31.02	76.71 ± 28.36	76.79 ± 31.93	t = -0.026	0.979
eGFR, mL/min	90.50 ± 25.15	92.46 ± 25.43	89.81 ± 25.04	t = 1.100	0.272
TC, mmol/L	5.24 ± 1.57	5.15 ± 1.49	5.27 ± 1.59	t = -0.809	0.419
TG, mmol/L	1.69 (1.15–2.72)	1.77 (1.15–2.88)	1.68 (1.15–2.63)	Z = -0.565	0.572
HDL-C, mmol/L	1.20 ± 0.49	1.20 ± 0.48	1.20 ± 0.49	t = 0.140	0.889
LDL-C, mmol/L	3.22 ± 1.13	3.18 ± 1.15	3.23 ± 1.13	t = -0.536	0.592
FPG, mmol/L	8.84 ± 3.14	8.43 ± 3.21	8.98 ± 3.10	t = -1.829	0.068
PPG, mmol/L	12.05 ± 4.04	11.88 ± 4.30	12.11 ± 3.94	t = -0.591	0.555
HbA1c, %	8.98 ± 2.45	8.79 ± 2.28	9.05 ± 2.51	t = -1.106	0.269
IR	4.03 (2.39–6.30)	3.46 (1.69–5.52)	4.29 (2.59–6.47)	Z = -3.056	0.002

Data are presented as mean ± standard deviation, median (interquartile range), or n (%) as appropriate. DSPN, distal symmetric polyneuropathy; DM, diabetes mellitus; SBP, systolic blood pressure; DBP, diastolic blood pressure; BMI, body mass index; WC, waist circumference; ALT, alanine aminotransferase; Ca, calcium; Cr, creatinine; eGFR, estimated glomerular filtration rate; TC, total cholesterol; TG, triglycerides; HDL-C, high-density lipoprotein cholesterol; LDL-C, low-density lipoprotein cholesterol; FPG, fasting plasma glucose; PPG, 2-hour postprandial plasma glucose; HbA1c, glycated hemoglobin; IR, insulin resistance.

### 3.2 Comparison of vitamin D levels between groups and subgroup analysis

25(OH)D and 25(OH)D_3_ concentrations were markedly reduced in the DSPN group relative to the non-DSPN group (23.95 ± 5.14 vs. 32.31 ± 5.97 ng/mL; 22.88 ± 5.11 vs. 31.19 ± 5.70 ng/mL) (P < 0.001). By contrast, 25(OH)D_2_ concentrations presented no evident intergroup difference (*P* = 0.645) (**Table 2**). Stratified analyses by gender, age, BMI, and season yielded similar results (**Supplementary Tables 2**–**5**).

**Table 2:** Comparison of serum vitamin D levels between the Non-DSPN and DSPN groups.

Variable	Total (n = 569)	Non-DSPN (n = 148)	DSPN (n = 421)	Statistic	P
25(OH)D_2_, ng/mL	0.79 (0.58–1.21)	0.75 (0.58–1.17)	0.80 (0.58–1.21)	Z = -0.461	0.645
25(OH)D_3_, ng/mL	25.04 ± 6.41	31.19 ± 5.70	22.88 ± 5.11	t = 16.483	<0.001
25(OH)D, ng/mL	26.12 ± 6.50	32.31 ± 5.97	23.95 ± 5.14	t = 16.302	<0.001

Data are presented as mean ± standard deviation, median (interquartile range), as appropriate. 25(OH)D, 25-hydroxyvitamin D; DSPN, distal symmetric polyneuropathy.

### 3.3 Trend analysis of vitamin D levels and the risk of DSPN

In quartile analysis, participants in Q4 exhibited a lower prevalence of DSPN and a reduced relative disease risk compared with Q1. Trend testing, with the median of each 25(OH)D quartile assigned as a dummy variable, further supported a graded negative association between serum 25(OH)D concentration and DSPN prevalence, with a significant P trend for ORs (P < 0.001) (**Table 3**).

**Table 3:** Prevalence and odds ratio of DSPN across serum 25(OH)D quartiles.

25(OH)D, ng/mL	n	Prevalence, %	OR (95% CI)	P
Q1 (8.71–22.10)	142	96	1.00 (reference)	/
Q2 (22.10–25.80)	141	95	0.85 (0.28–2.58)	0.767
Q3 (25.80–29.70)	143	75	0.13 (0.05–0.32)	<0.001
Q4 (29.70–56.60)	143	31	0.02 (0.01–0.05)	<0.001
P trend for ORs				<0.001

25(OH)D, 25-hydroxyvitamin D; DSPN, distal symmetric polyneuropathy; OR, odds ratio; CI, confidence interval.

### 3.4 Relation of the level of 25(OH)D to MNSI scores

MNSI-Q and MNSI-PE scores were significantly higher among patients with DSPN compared to those without DSPN (P < 0.001) (**Table 4**). Both the MNSI-Q and MNSI-PE scores decreased gradually with the rising 25(OH)D concentrations (P < 0.001) (**Supplementary Table 6**).

**Table 4:** Comparison of michigan neuropathy screening instrument scores between the Non-DSPN and DSPN groups.

Variable	Total (n = 569)	Non-DSPN (n = 148)	DSPN (n = 421)	Statistic	P
MNSI-Q	2.00 (1.00–5.00)	0.00 (0.00–1.00)	4.00 (1.00–6.00)	Z = -13.802	<0.001
MNSI-PE	2.00 (1.00–4.00)	0.00 (0.00–0.00)	3.00 (2.00–4.00)	Z = -16.693	<0.001

Data are presented as median (interquartile range). DSPN, distal symmetric polyneuropathy; MNSI-Q, Michigan Neuropathy Screening Instrument Questionnaire; MNSI-PE, Michigan Neuropathy Screening Instrument Physical Examination.

### 3.5 Logistic regression analysis of factors associated with DSPN

**Figure 2:**
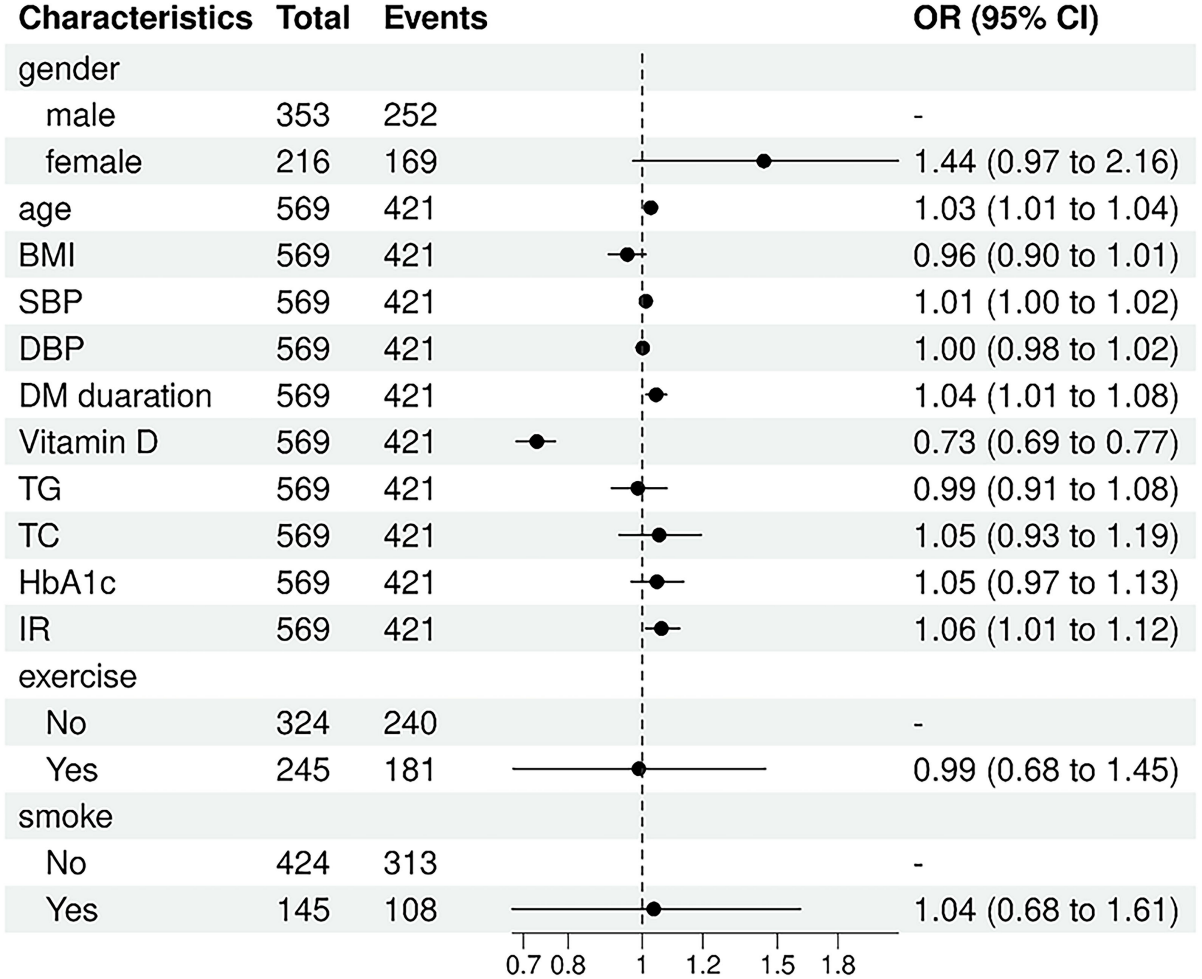
Forest plot of univariable logistic regression analysis for factors associated with DSPN. OR, odds ratio; CI, confidence interval; DSPN, distal symmetric polyneuropathy; BMI, body mass index; SBP, systolic blood pressure; DBP, diastolic blood pressure; TG, triglycerides; TC, total cholesterol; HbA1c, glycated hemoglobin A1c; IR, insulin resistance.

According to the above analyses and past literature, 13 possible risk factors for DSPN were incorporated in the analysis. The Pearson correlation matrix showed partial biological relationships between various variables, such as DM duration and age, SBP and DBP, TC and TG, IR and TG, and smoking status and gender, with no strong correlations detected (**Supplementary Figure 1**). This was further supported by variance inflation factor analysis, in which all VIF values were less than 2 (**Supplementary Figure 2**). In univariable logistic regression, age (OR = 1.03, 95% CI: 1.01–1.04, *P* < 0.01), DM duration (OR = 1.04, 95% CI: 1.01–1.07, *P* < 0.001), and IR (OR = 1.06, 95% CI: 1.01–1.11, *P* < 0.05) may serve as risk factors for predicting the occurrence of DSPN. Conversely, a negative relationship emerged between 25(OH)D and DSPN prevalence, with evidence suggesting a protective role against the initiation of DSPN (OR = 0.73, 95% CI: 0.69–0.77, P < 0.001) (**Figure 2**) (**Supplementary Table 7**).

**Figure 3:**
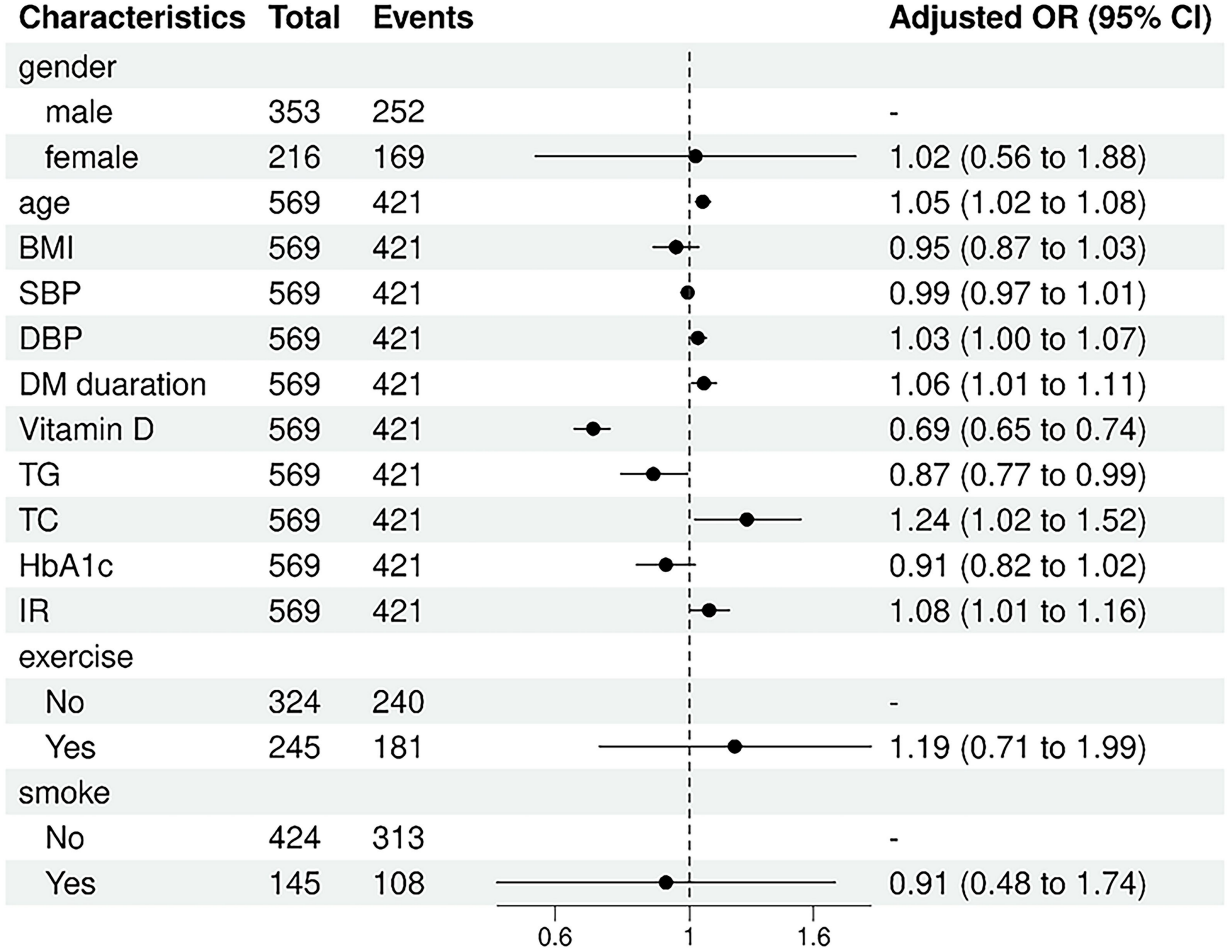
Forest plot of multivariable logistic regression analysis for factors associated with DSPN. OR, odds ratio; CI, confidence interval; DSPN, distal symmetric polyneuropathy; BMI, body mass index; SBP, systolic blood pressure; DBP, diastolic blood pressure; TG, triglycerides; TC, total cholesterol; HbA1c, glycated hemoglobin A1c; IR, insulin resistance.

A multivariate logistic regression model was developed to make corrections on potential confounding variables. Independent variables included classic DSPN risk factors, and the parameters which were significant in the univariate regression. Modifications indicated that age, diabetes duration, TG, TC, and IR did not have a significant interaction with the risk of DSPN, but 25(OH)D was a stable independent protective factor (**Figure 3**) (**Supplementary Table 8**).

Sequentially adjusted multivariable models showed that the correlation between 25(OH)D and DSPN remained robust after progressive adjustment for potential confounders. The odds ratio for 25(OH)D (OR = 0.69, 95% CI: 0.65–0.74, P < 0.001) decreased further, indicating a significant protective effect (**Table 5**).

**Table 5:** Analysis of DSPN risk factors for confounding bias controlled by multiple models.

Variable	Model 1		Model 2		Model 3		Model 4	
	OR (95% CI)	P	OR (95% CI)	P	OR (95% CI)	P	OR (95% CI)	P
25(OH)D, ng/mL	0.73 (0.69–0.77)	<0.001	0.72 (0.67–0.76)	<0.001	0.71 (0.67–0.76)	<0.001	0.69 (0.65–0.74)	<0.001

Model 1 was unadjusted. Model 2 was adjusted for age, BMI, and gender. Model 3 was adjusted for age, BMI, gender, smoke, and exercise. Model 4 was adjusted for age, BMI, gender, smoke, exercise, SBP, DBP, DM duration, TG, TC, HbA1c, and IR. 25(OH)D, 25-hydroxyvitamin D; DSPN, distal symmetric polyneuropathy; OR, odds ratio; CI, confidence interval; BMI, body mass index; SBP, systolic blood pressure; DBP, diastolic blood pressure; DM, diabetes mellitus; TG, triglycerides; TC, total cholesterol; HbA1c, glycated hemoglobin; IR, insulin resistance.

### 3.6 PSM analysis

#### 3.6.1 Baseline balance before and after matching

After 1:1 propensity score matching, the DSPN and non-DSPN groups achieved good balance in baseline characteristics, including age, gender, BMI, and DM duration (**Table 6**). The standardized mean differences (SMDs) were reduced to less than 0.1 after matching, as shown in the SMD distribution plot (**Supplementary Figure 3**). The Love plot further demonstrated improved covariate balance between the two groups after matching (**Supplementary Figure 4**).

**Table 6:** Baseline characteristics before and after propensity score matching in the Non-DSPN and DSPN groups.

Variable	Before PSM						After PSM					
	Total (n = 569)	Non-DSPN (n = 148)	DSPN (n = 421)	Statistic	P	SMD	Total (n = 286)	Non-DSPN (n = 143)	DSPN (n = 143)	Statistic	P	SMD
Age, years	58.36 ± 11.38	55.87 ± 11.44	59.24 ± 11.24	t = -3.117	0.002	0.299	56.43 ± 11.28	56.35 ± 11.23	56.50 ± 11.37	t = -0.115	0.908	0.014
BMI, kg/m²	24.13 ± 3.36	24.51 ± 3.41	24.00 ± 3.34	t = 1.585	0.114	-0.15	24.21 ± 3.45	24.36 ± 3.37	24.05 ± 3.52	t = 0.771	0.442	-0.09
SBP, mmHg	129.35 ± 17.39	127.05 ± 16.46	130.16 ± 17.66	t = -1.871	0.062	0.176	126.89 ± 16.72	127.48 ± 16.40	126.30 ± 17.07	t = 0.597	0.551	-0.07
DBP, mmHg	81.06 ± 10.96	80.94 ± 10.40	81.10 ± 11.17	t = -0.155	0.877	0.015	80.69 ± 10.49	81.02 ± 10.48	80.35 ± 10.52	t = 0.540	0.589	-0.06
HbA1c, %	8.98 ± 2.45	8.79 ± 2.28	9.05 ± 2.51	t = -1.106	0.269	0.103	8.89 ± 2.44	8.78 ± 2.29	9.00 ± 2.59	t = -0.750	0.454	0.084
TC, mmol/L	5.24 ± 1.57	5.15 ± 1.49	5.27 ± 1.59	t = -0.809	0.419	0.076	5.22 ± 1.58	5.18 ± 1.50	5.25 ± 1.65	t = -0.397	0.692	0.045
DM duration, years	7.00 (2.00–10.17)	5.00 (1.00–10.00)	8.00 (3.00–11.00)	Z = -3.074	0.002	0.255	6.00 (1.00–10.00)	5.00 (1.00–10.00)	6.00 (2.00–10.00)	Z = -0.786	0.432	0.028
TG, mmol/L	1.69 (1.15–2.72)	1.77 (1.15–2.88)	1.68 (1.15–2.63)	Z = -0.565	0.572	-0.03	1.71 (1.10–2.79)	1.68 (1.11–2.77)	1.72 (1.10–2.79)	Z = -0.226	0.821	0.074
IR	4.03 (2.39–6.30)	3.46 (1.69–5.52)	4.29 (2.59–6.47)	Z = -3.056	0.002	0.19	3.57 (1.99–5.40)	3.41 (1.68–5.62)	3.68 (2.38–5.27)	Z = -0.745	0.456	-0.09
Gender, n (%)				χ² = 3.270	0.071					χ² = 0.063	0.801	
Male	353 (62.04)	101 (68.24)	252 (59.86)			-0.17	192 (67.13)	97 (67.83)	95 (66.43)			-0.03
Female	216 (37.96)	47 (31.76)	169 (40.14)			0.17	94 (32.87)	46 (32.17)	48 (33.57)			0.03
Exercise, n (%)				χ² = 0.003	0.958					χ² = 0.014	0.905	
No	324 (56.94)	84 (56.76)	240 (57.01)			0.005	163 (56.99)	82 (57.34)	81 (56.64)			-0.01
Yes	245 (43.06)	64 (43.24)	181 (42.99)			-0.005	123 (43.01)	61 (42.66)	62 (43.36)			0.01
Smoke, n (%)				χ² = 0.025	0.875					χ² = 0.000	1.000	
No	424 (74.52)	111 (75.00)	313 (74.35)			-0.015	214 (74.83)	107 (74.83)	107 (74.83)			0
Yes	145 (25.48)	37 (25.00)	108 (25.65)			0.015	72 (25.17)	36 (25.17)	36 (25.17)			0

Data are presented as mean ± standard deviation, median (interquartile range), or n (%) as appropriate. DSPN, distal symmetric polyneuropathy; PSM, propensity score matching; SMD, standardized mean difference; BMI, body mass index; SBP, systolic blood pressure; DBP, diastolic blood pressure; HbA1c, glycated hemoglobin; TC, total cholesterol; DM, diabetes mellitus; TG, triglycerides; IR, insulin resistance.

#### 3.6.2 Multivariable analysis to address residual confounding after PSM

Multivariable analysis was performed in the matched sample to further reduce confounding bias. Even after full adjustment, 25(OH)D remained independently associated with lower DSPN risk (OR = 0.67, 95% CI: 0.61–0.74, *P* < 0.001) (**Table 7**). Compared with the pre-PSM analysis, 25(OH)D showed a lower OR after full adjustment, suggesting a stronger protective effect against DSPN.

**Table 7:** Analysis of DSPN risk factors for confounding bias in data multi-model control after PSM.

Variables	Model 1		Model 2		Model 3		Model 4	
	OR (95% CI)	P	OR (95% CI)	P	OR (95% CI)	P	OR (95% CI)	P
25(OH)D, ng/mL	0.70 (0.64–0.76)	<0.001	0.69 (0.63–0.75)	<0.001	0.68 (0.63–0.75)	<0.001	0.67 (0.61–0.74)	<0.001

Model 1 was unadjusted. Model 2 was adjusted for age, BMI, and gender. Model 3 was adjusted for age, BMI, gender, smoke, and exercise. Model 4 was adjusted for age, BMI, gender, exercise, smoke, SBP, DBP, DM duration, TG, TC, HbA1c, and IR. 25(OH)D, 25-hydroxyvitamin D; DSPN, distal symmetric polyneuropathy; OR, odds ratio; CI, confidence interval; PSM, propensity score matching; BMI, body mass index; SBP, systolic blood pressure; DBP, diastolic blood pressure; DM, diabetes mellitus; TG, triglycerides; TC, total cholesterol; HbA1c, glycated hemoglobin; IR, insulin resistance.

### 3.7 RCS analysis

**Figure 4:**
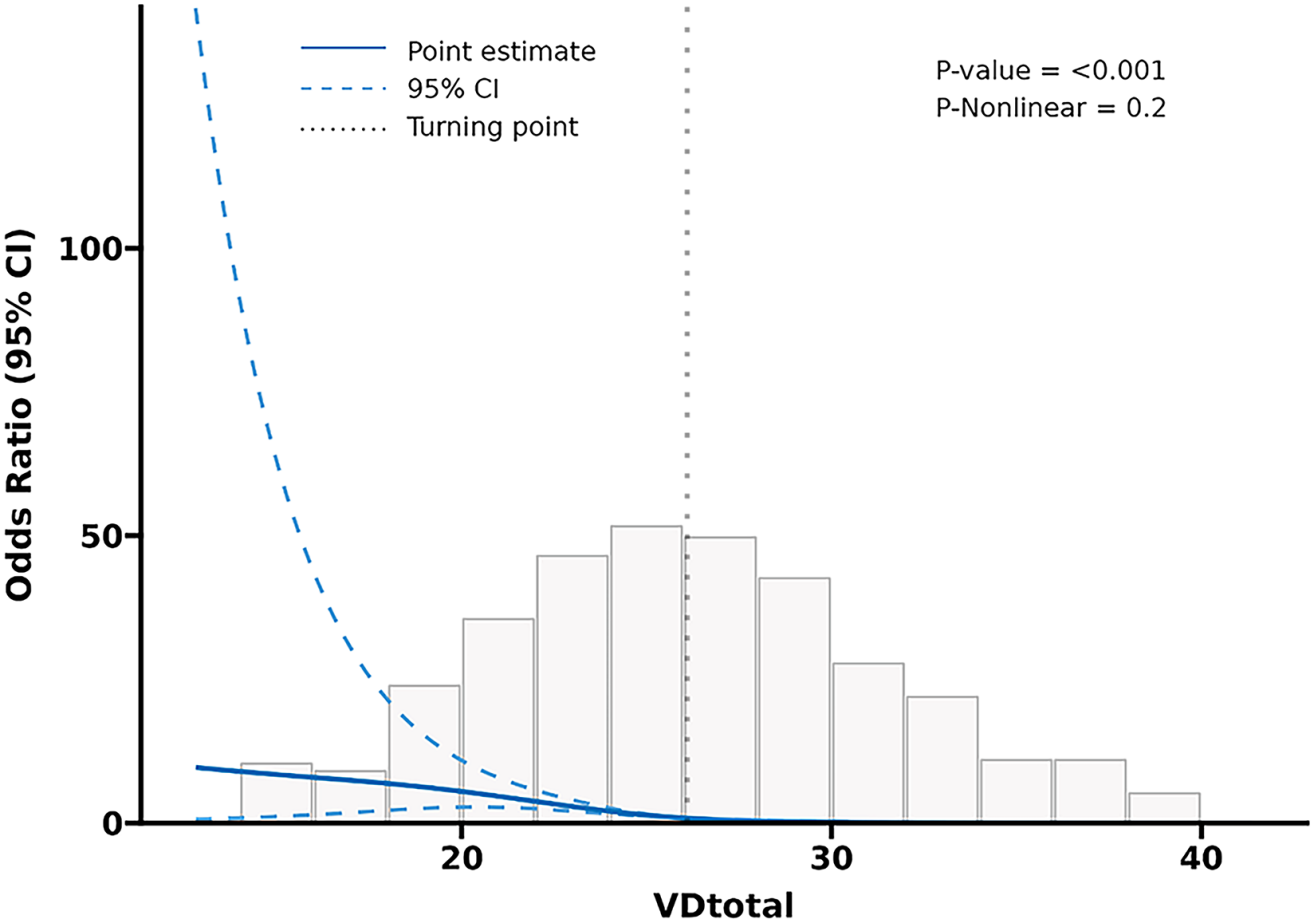
Restricted cubic spline analysis exploring the relationship between serum 25(OH)D levels and the risk of DSPN. The model was adjusted for age, diabetes duration, triglycerides, total cholesterol, and insulin resistance. P for nonlinearity, 0.20. 25(OH)D, 25-hydroxyvitamin D; DSPN, distal symmetric polyneuropathy.

**Figure 5:**
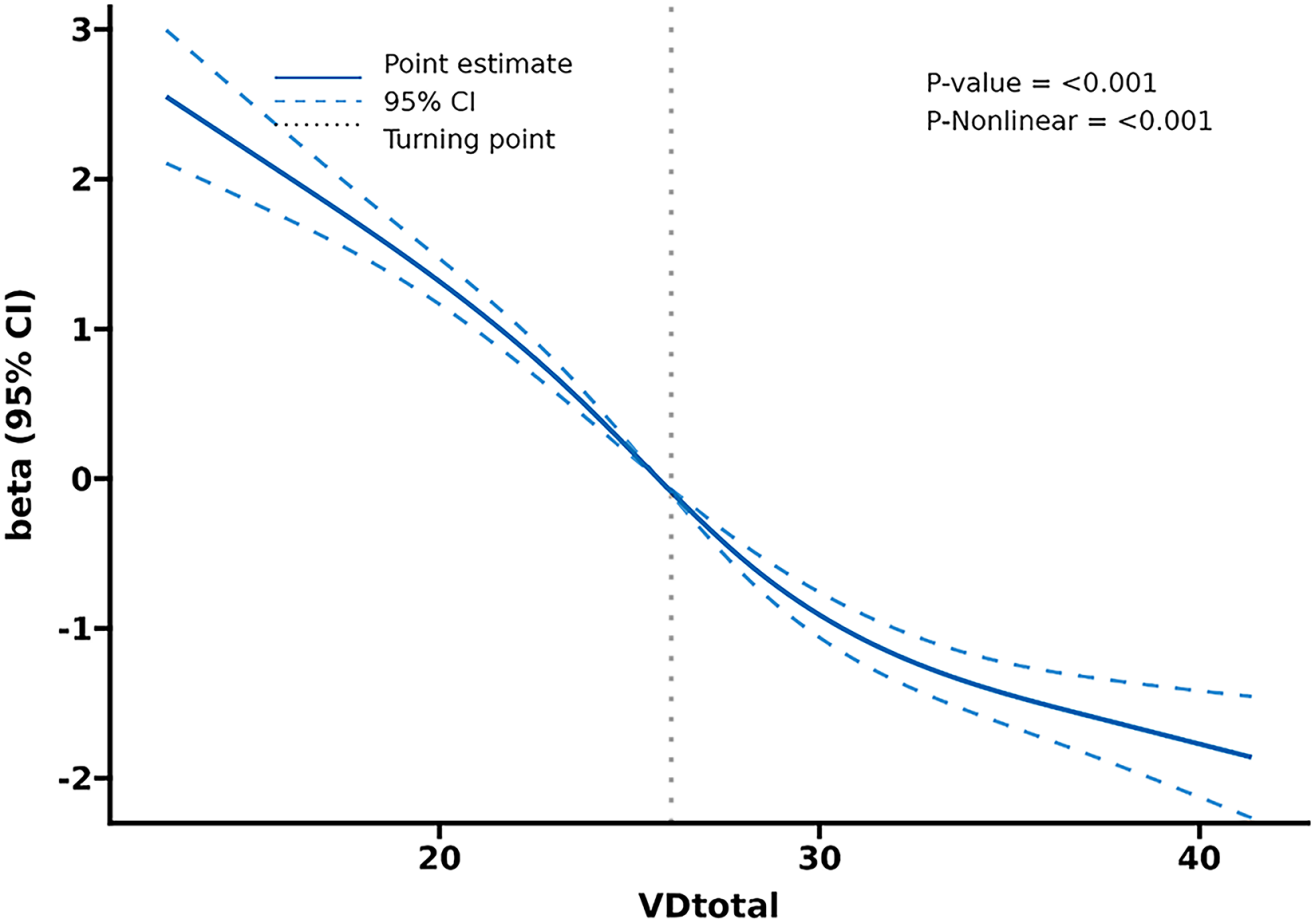
Restricted cubic spline analysis exploring the relationship between serum 25(OH)D levels and DSPN severity assessed by MNSI-PE score. The model was adjusted for age, diabetes duration, triglycerides, total cholesterol, and insulin resistance. A significant nonlinear correlation was observed between 25(OH)D and MNSI-PE, with an obvious turning point at 26.1 ng/mL. 25(OH)D, 25-hydroxyvitamin D; MNSI-PE, Michigan Neuropathy Screening Instrument–Physical Examination.

RCS analysis confirmed a negative correlation between 25(OH)D concentrations and DSPN risk (P for nonlinearity = 0.20, P < 0.01) (**Figure 4**). Further RCS analysis revealed a nonlinear association between 25(OH)D levels and neuropathy severity assessed by MNSI-PE, with a specific turning point around 26.1 ng/mL (**Figure 5**). Based on this turning point, participants were stratified into two groups: those with 25(OH)D below 26.1 ng/mL and those at or above this level. Piecewise linear regression revealed that the protective effect was more robust under the turning point (β= −0.50), whereas the effect size declined at concentrations above this threshold (β = −0.35) (**Supplementary Table 9**).

## 4 Discussion

The research, which was focused on a multicenter cross-sectional design of observation, involved the patients with T2DM in Fujian, China, and carried out the systematic investigation of the connection between 25(OH)D levels and the risk and severity of DSPN. The main findings were as follows: (1) total 25(OH)D and 25(OH)D_3_ levels were markedly reduced in the DSPN patients and the difference persisted after the stratification by gender, age and other factors while no group difference was observed for 25(OH)D_2_; (2) 25(OH)D was negatively related with the predisposition to DSPN and remained an independent protective factor after the adjustment of the multivariate analysis; (3) The dose-response relationship was found to be significant and not linear: the turning point was set at the range of approximately 26.1 ng/mL, below which the risk of DSPN increased significantly.

The prevalence of DSPN in this cohort was high, reaching 73.99%. This observation accorded with a previous study undertaken in Shanghai, China, where the prevalence of DSPN was reported to be 61.8% (26). A 25-year foreign cohort study reported that the prevalence of clinically diagnosed DSPN was around 45%, while studies adopting more sensitive diagnostic approaches reported prevalence figures ranging from 60% to 75% (7, 27). Collectively, these data reveal that DSPN is prevalent globally without regional or population restrictions, and discrepancies in prevalence across studies are mainly attributable to inconsistent diagnostic criteria. Importantly, many patients with DSPN lack typical symptoms in the early stages, and nerve damage is often irreversible once the disease becomes clinically apparent. Therefore, the identification of indicators that enable early detection and timely intervention carries significant clinical relevance.

Patients in the DSPN group in our study were generally older than those who did not develop DSPN. Furthermore, age was identified as an independent determinant of DSPN, based on the results of statistical modeling consistent with findings from a previous observational study in China (28). Accumulated evidence has demonstrated that the prevalence of DPN climbed gradually alongside aging among T2DM populations (29). Furthermore, age not only has a direct impact on DSPN development, but also is recognized as an important determinant which can influence vitamin D status. The concentration of 7-dehydrocholesterol in the skin has been reported to decline by more than 50% between 20 and 80 years of age. Chalcraft et al. further showed that vitamin D levels decrease by about 13% per decade, while cutaneous synthesis of vitamin D_3_ in individuals aged 70 is approximately 50% of that in those aged 20 (30). Hence, age was regarded as a potential confounding factor in the stratified analysis, and the observed association remained persistent. Even after accounting for this factor, patients with DSPN continued to have lower 25(OH)D levels than those without DSPN, indicating a robust relationship. Because vitamin D synthesis is influenced by environmental factors, several related variables—including sun exposure, season, latitude, skin color, and sun protection practices—were assessed in this study. The distribution of these factors did not differ significantly between groups, indicating a comparable environmental background with respect to determinants of vitamin D status. This implies that significant environmental changes on vitamin D status were more or less similar in the two groups, which contributed to the increased internal validity of the study. Factors including age and season were considered in the adjusted analyses; however, the relationship between lower vitamin D levels and DSPN remained. Moreover, patients with DSPN also tended to have a longer DM duration, and DM duration was determined as a risk factor on its own. Though age and diabetes duration are not subject to change, their consistent relationship with DSPN highlights the significance of screening and early intervention in T2DM patients.

Several mechanisms may underlie the observed association. Apart from its well-known function in calcium and phosphate homeostasis, vitamin D participates in immune regulation, inflammatory signaling, oxidative stress modulation, lipid metabolism, promotion of cell differentiation and proliferation, and reduction of cytokine storms, as well as neurotrophic support, through the vitamin D receptor pathway (31–36). These processes suggest that when vitamin D status is sufficient, both the risk of DSPN development and the rate of its subsequent progression may be reduced.

DSPN patients possessed low levels of total 25(OH)D and 25(OH)D_3_ in comparison to the non-DSPN group with no differences in 25(OH)D_2_ levels. A previous cross-sectional study of Shanghai revealed the same findings (37). Quartile analysis further demonstrated that participants in the highest two 25(OH)D quartiles (Q3, Q4) had a notably lower risk of developing DSPN. By contrast, little statistical divergence could be found among the bottom two quartiles (Q1 and Q2). Specifically, when serum 25(OH)D concentration reached 25.8 ng/mL, the risk of DSPN was reduced by 87%. Subsequent trend test results showed that DSPN occurrence declined progressively with increasing 25(OH)D concentrations. In addition, MNSI-PE scores tended to drop as serum 25(OH)D concentrations rose. Obvious disparities existed across the four subgroups, fully demonstrating that higher 25(OH)D levels may confer protective benefits against DSPN conditions. This finding agreed well with the outcomes from a contemporary investigation using the UK Biobank database, which enrolled 14,709 individuals with T2DM and no baseline DSPN. During the 11.2-year follow-up period, Cox regression analysis revealed that those who had adequate vitamin D levels had a 52% less chance of developing DSPN (38). This observation highly suggests that the optimal nutritional status of vitamin D is an important protective factor towards the prevention of the development of DSPN.

Univariate logistic regression analysis, using DSPN as the study endpoint, identified a negative association between serum 25(OH)D and DSPN (OR = 0.73). After controlling for relevant confounding factors, multivariate regression further demonstrated that vitamin D possessed an independent linkage to DSPN and presented obvious protective properties (OR = 0.69). This relationship was still clear in the following multivariable analyses (OR = 0.67) after propensity score matching. Interestingly, additional modification of conventional risk factors did not lessen this protective association; rather, the strength of the association exhibited a minor increase. The uniformity of the results among all of the models indicates that higher vitamin D levels confer a clear protective effect against DSPN, while vitamin D insufficiency may raise individual susceptibility to DSPN. We also found that our findings matched a previously published meta-analysis carried out by Qu et al. In this meta-analysis, 1,484 individuals with T2DM were included in 6 independent studies, and confirmed that vitamin D insufficiency alone could increase the risk of developing DSPN (39).

RCS analysis further demonstrated a significant nonlinear relationship linking serum 25(OH)D concentrations to MNSI-PE scores, a finding that echoes previous evidence by a prior case–control investigation (40). A turning point was found to be at about 26.1 ng/mL, which is near the 25.8 ng/mL value in the quartile analysis, at which a significant protective effect was first seen in the Q3 group. The gradient of the curve was steeper below this point and more gradual at higher concentrations. In line with this, the absolute value of the β coefficient at the turning point declined with a change from -0.50 to -0.35, which shows that the association is stronger when vitamin D levels are low. Collectively, these observations indicate that reduced vitamin D levels correlate with more severe DSPN. Likewise, research carried out in Wenzhou yielded comparable findings, showing higher DSPN odds in subjects with suboptimal vitamin D status (41). On a global scale, vitamin D deficiency affects approximately one billion individuals, constituting a substantial public health burden. The US Endocrine Society guidelines on vitamin D nutrition categorized the amount of vitamin D into three groups: sufficiency (≥30 ng/mL), insufficiency (20–30 ng/mL), and deficiency (<20 ng/mL) (42–44). However, based on our findings, the conventional level of vitamin D deficiency can be reassessed in patients with DSPN and the 25(OH)D 26.1 ng/mL cutoff threshold might be a more appropriate cutoff level in DSPN patients allowing earlier warning and treatment for more patients. It was also discovered that the slope of the curve was not as steep with the 25(OH)D concentrations exceeding 26.1 ng/mL, indicating that rising vitamin D concentrations did not play a role in causing significant changes in MNSI-PE scores beyond this level. This meant that the protective effect that vitamin D has may be greater at lower concentrations in the blood.

It is worth noting that neither FBG nor HbA1c was independently associated with DSPN risk in this study. This finding corresponds with findings of large-scale clinical trial studies such as ACCORD that have shown intensive glycemic control in isolation has only limited efficacy in preventing DSPN. Although glycemic abnormality is a common feature of both T1DM and T2DM, glucose-lowering interventions exert markedly different effects on the incidence of DSPN between the two populations. Intensive glycemic control can reduce the risk of DSPN by 78% in patients with T1DM (45), whereas patients with T2DM gain minimal benefits. Multiple studies have confirmed that glucose-lowering monotherapy only slightly cuts the risk of DSPN among individuals with T2DM, with a relative risk reduction of merely 5% to 9% (46). This discrepancy is presumed to be closely associated with the frequent coexistence of metabolic syndrome components such as obesity and insulin resistance in T2DM. Furthermore, most patients with T2DM have experienced impaired glucose tolerance for several years prior to formal diagnosis, during which peripheral nerve damage may have already developed. This fact may explain why glycated hemoglobin failed to emerge as an independent predictor of DSPN in the present study, and also indicates that vitamin D status could serve as a novel intervention target for DSPN in patients with T2DM.

### 4.1 Strengths and limitations

There are certain strengths associated with this study. Firstly, it was founded on a sufficient number of cohort that was registered in different clinical centers. Secondly, concentrations were determined by the LC-MS/MS, which is more specific as compared to the conventional immunoassays. In addition, our findings suggest that some individuals who have DSPN might require an increased amount of vitamin D. The lower concentrations, below 26.1 ng/mL, directly predict higher DSPN susceptibility and greater disease severity. Additionally, sensitivity analysis, stratified analysis, PSM, and RCS analysis were also performed which strengthened the findings.

Several limitations exist in this study. First, owing to the cross-sectional study design, it is unable to establish definite causal relationships, leaving reverse causation as a plausible possibility. Second, despite our extensive adjustments for known confounders, the observational nature of this study means that residual confounding from unmeasured factors (such as genetic predisposition) cannot be completely excluded. Third, the sample, since the participants were recruited in a single region of China, might not be applicable to other populations.

### 4.2 Future work

Further large-scale, multicenter, prospective randomized controlled trials of the diverse population of T2DM patients are needed to validate the relationship between vitamin D and DSPN. These studies should also focus on identifying optimal cutoff values for 25(OH)D for clinical evaluation and evidence-based supplementation guidelines to prevent or delay the onset and progression of DSPN.

## 5 Conclusion

Lower serum 25(OH)D levels are associated with an increased risk of DSPN and exhibit a nonlinear relationship with neuropathy severity. Notably, the severity of DSPN appears to accelerate markedly when 25(OH)D levels fall below 26.1 ng/mL.

## Data Availability

The original contributions presented in the study are included in the article/**Supplementary Material**. Further inquiries can be directed to the corresponding author.

## Supplementary material

Supplementary Figure 1 Rectangular graph of correlation coefficients between independent variables. Color intensity represents the strength of the correlation coefficient.

Supplementary Figure 2 Variance inflation factor (VIF) for collinearity diagnostics of independent variables. All variables showed VIF values < 2, indicating no significant multicollinearity.

Supplementary Figure 3 Standardized mean differences (SMD) of covariates before and after propensity score matching (PSM). SMD values < 0.1 indicate good balance between groups.

Supplementary Figure 4 Love plot illustrating covariate balance before and after propensity score matching (PSM). Covariates were well balanced after matching.
