# Supplementary figures and images for: Lower serum 25-hydroxyvitamin D levels predict higher risk of DSPN in type 2 diabetes, and exhibit a non-linear association with the severity of DSPN
Source: Front Endocrinol (Lausanne). 2026 Jul 13;17:1890018. doi: 10.3389/fendo.2026.1890018 (PMC13402170; doi:10.3389/fendo.2026.1890018)

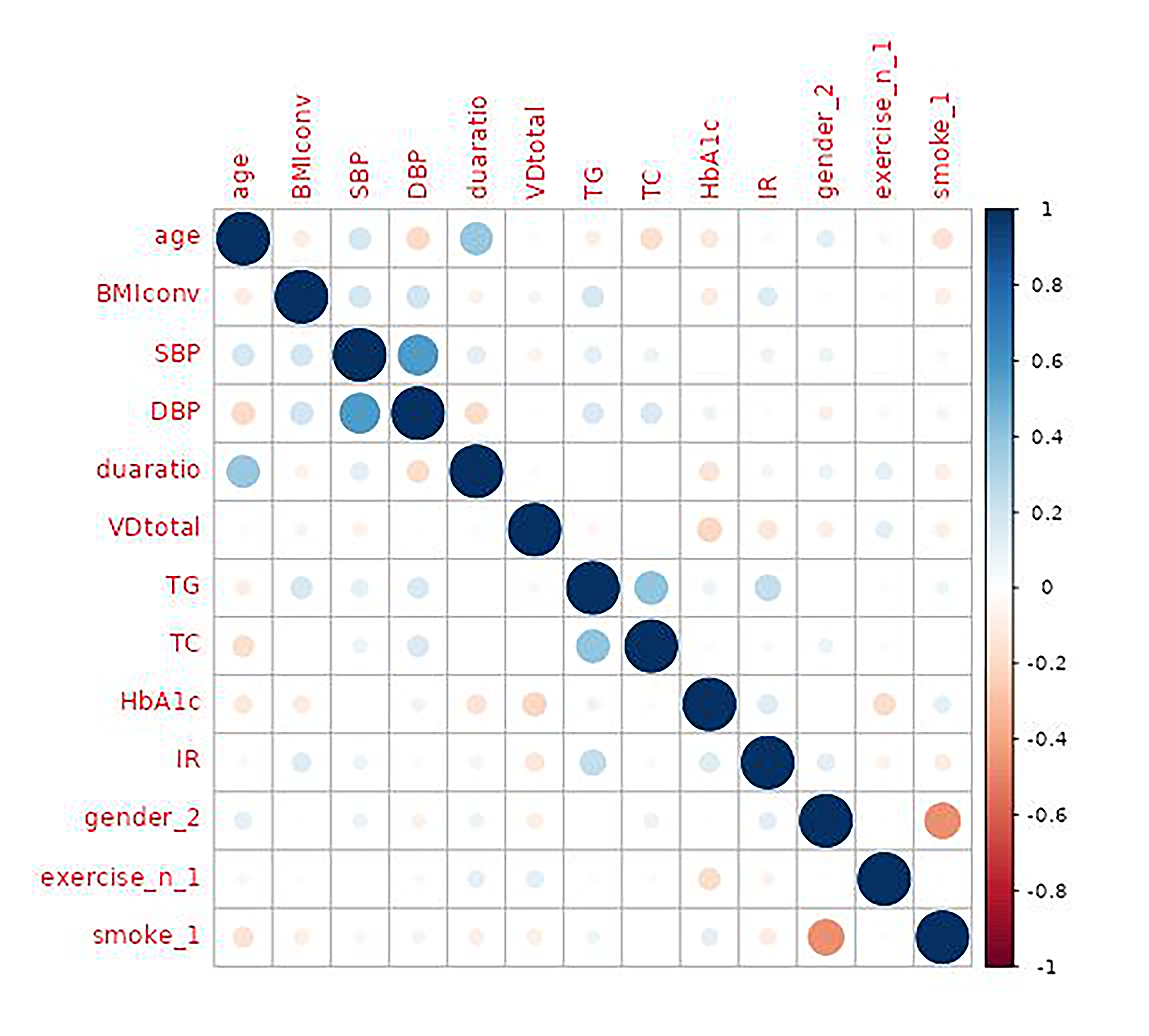

Supplement: Supplementary Figure 1 — Rectangular graph of correlation coefficients between independent variables. Color intensity represents the strength of the correlation coefficient. [file Image1.tif]

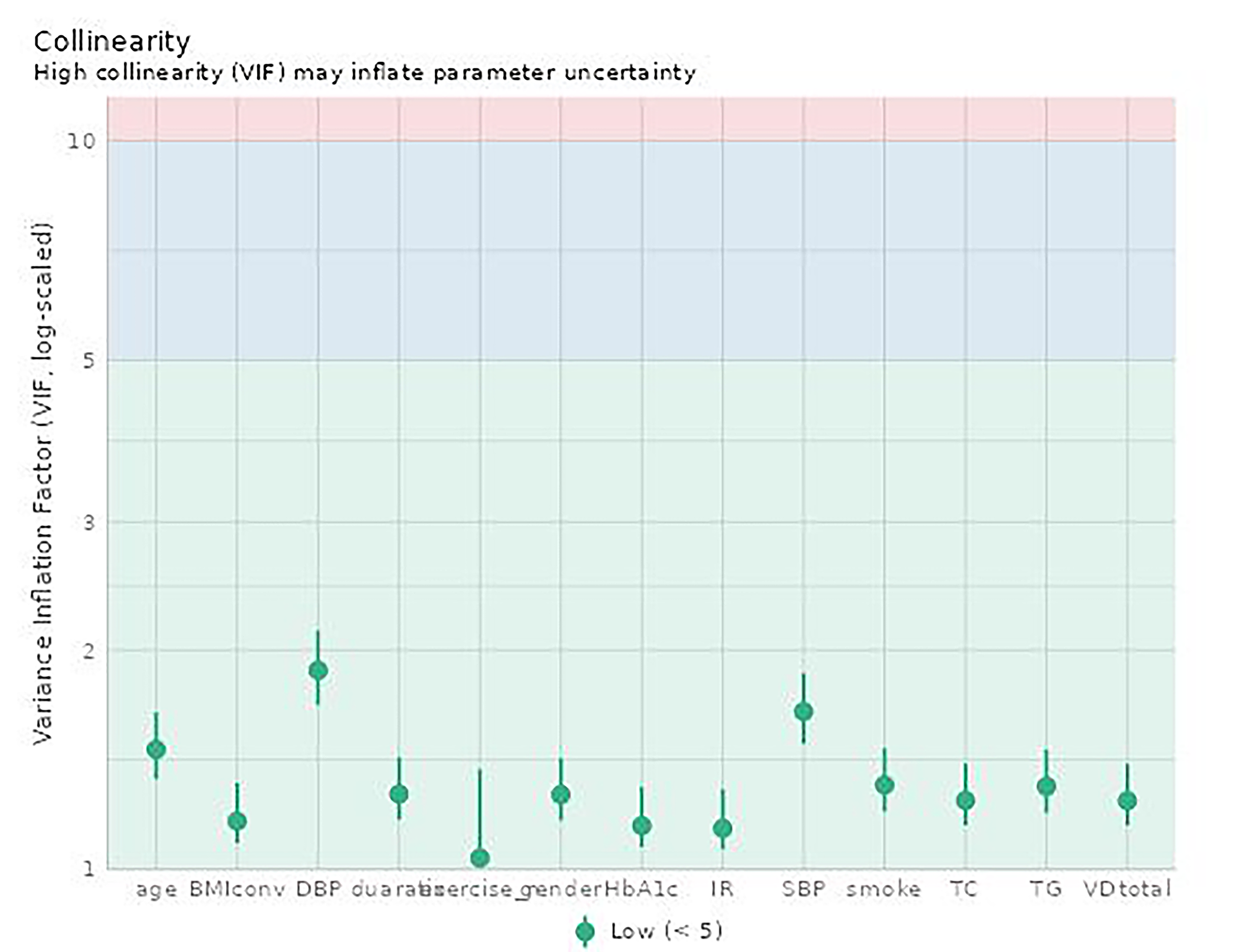

Supplement: Supplementary Figure 2 — Variance inflation factor (VIF) for collinearity diagnostics of independent variables. All variables showed VIF values < 2, indicating no significant multicollinearity. [file Image2.tif]

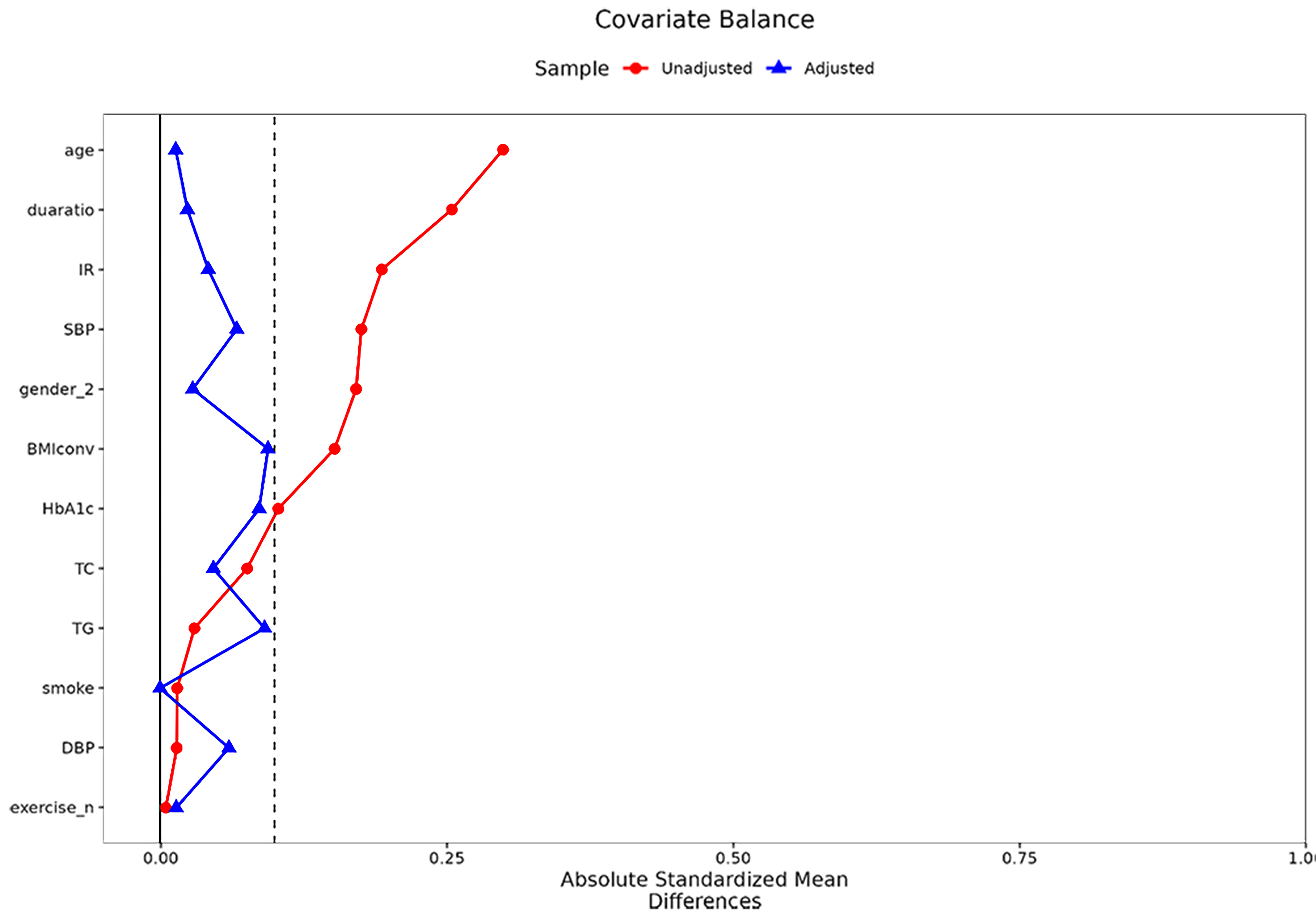

Supplement: Supplementary Figure 3 — Standardized mean differences (SMD) of covariates before and after propensity score matching (PSM). SMD values < 0.1 indicate good balance between groups. [file Image3.tif]

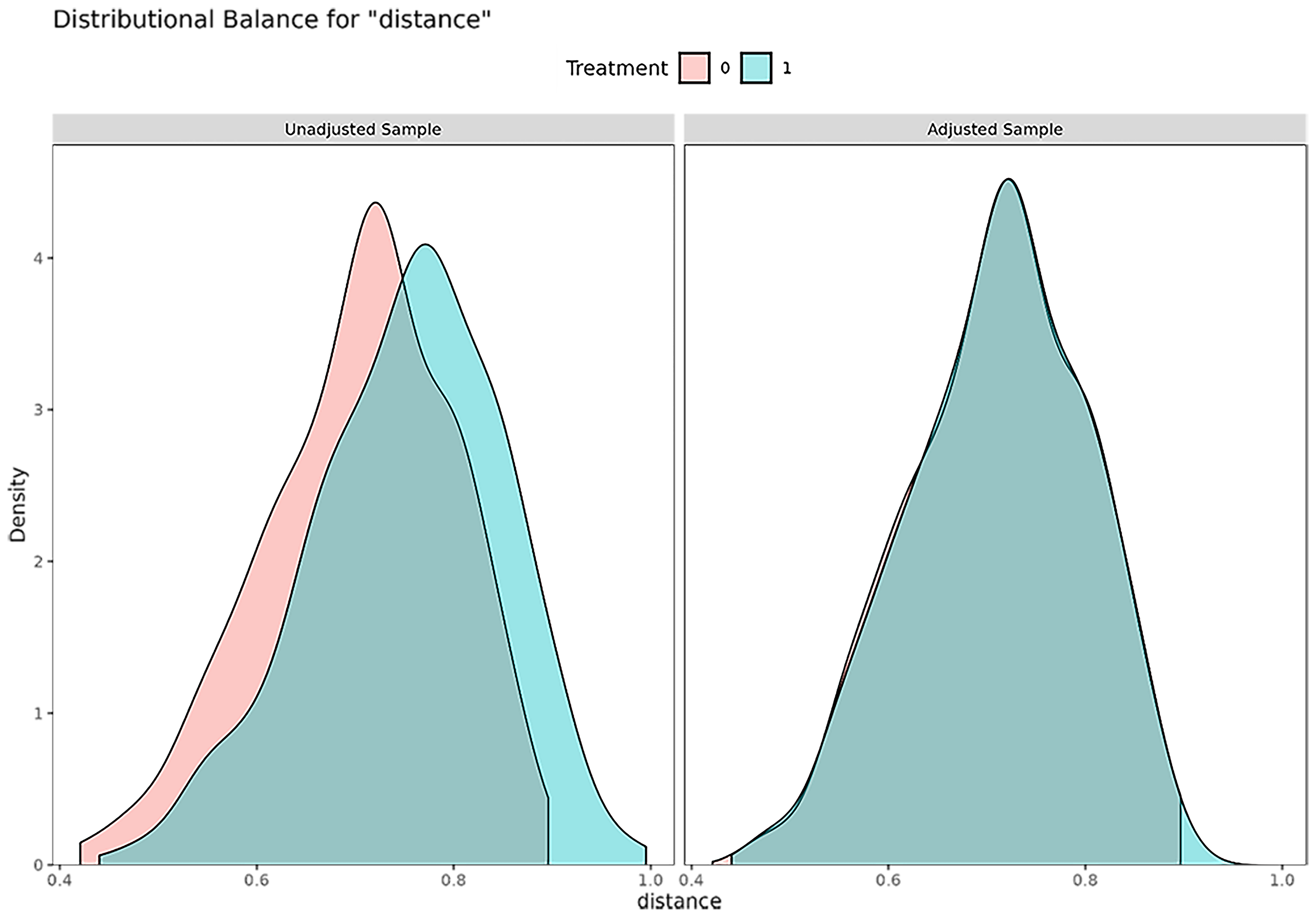

Supplement: Supplementary Figure 4 — Love plot illustrating covariate balance before and after propensity score matching (PSM). Covariates were well balanced after matching. [file Image4.tif]
