# Supplementary material for: Lower serum 25-hydroxyvitamin D levels predict higher risk of DSPN in type 2 diabetes, and exhibit a non-linear association with the severity of DSPN
Source: Front Endocrinol (Lausanne). 2026 Jul 13;17:1890018. doi: 10.3389/fendo.2026.1890018 (PMC13402170; doi:10.3389/fendo.2026.1890018)
Supplement: Supplementary file 5 [file SupplementaryFile1.docx]

**Table S1. Comparison of natural environmental factors between the Non-DSPN and DSPN groups.**

| Variable | Total (n = 569) | Non-DSPN (n = 148) | DSPN (n = 421) | Statistic | *P* value |
| --- | --- | --- | --- | --- | --- |
| Sun exposure, n (%) |  |  |  | χ² = 2.971 | 0.085 |
| No | 41 (7.21) | 35 (8.31) | 6 (4.05) |  |  |
| Yes | 528 (92.79) | 386 (91.69) | 142 (95.95) |  |  |
| Use of sun-protective clothing, n (%) |  |  |  | χ² = 0.182 | 0.669 |
| No | 531 (93.32) | 394 (93.59) | 137 (92.57) |  |  |
| Yes | 38 (6.68) | 27 (6.41) | 11 (7.43) |  |  |
| Use of sunscreen, n (%) |  |  |  | χ² = 0.000 | 1.000 |
| No | 554 (97.36) | 410 (97.39) | 144 (97.30) |  |  |
| Yes | 15 (2.64) | 11 (2.61) | 4 (2.70) |  |  |
| Skin tone, n (%) |  |  |  | - | 0.394 |
| Lighter than normal | 13 (2.28) | 10 (2.38) | 3 (2.03) |  |  |
| Normal | 538 (94.55) | 395 (93.82) | 143 (96.62) |  |  |
| Darker than normal | 18 (3.16) | 16 (3.80) | 2 (1.35) |  |  |
| Season, n (%) |  |  |  | χ² = 2.578 | 0.108 |
| Winter–Spring | 259 (45.52) | 200 (47.51) | 59 (39.86) |  |  |
| Summer–Autumn | 310 (54.48) | 221 (52.49) | 89 (60.14) |  |  |

Data are presented as n (%).

Abbreviations: DSPN = distal symmetric polyneuropathy.

**Table S2 Comparison of vitamin D levels stratified by sex.**

| Variable | Male (n = 353) | | | Female (n = 216) | | |
| --- | --- | --- | --- | --- | --- | --- |
|  | Non-DSPN (n = 101) | DSPN (n = 252) | *P* value | Non-DSPN (n = 47) | DSPN (n = 169) | *P* value |
| 25(OH)D, ng/mL | 32 ± 6 | 24 ± 5 | <0.001 | 32 ± 5 | 24 ± 5 | <0.001 |
| 25(OH)D₂, ng/mL | 0.72 (0.52–1.10) | 0.79 (0.59–1.13) | 0.246 | 0.86 (0.66–1.44) | 0.85 (0.54–1.29) | 0.357 |
| 25(OH)D₃, ng/mL | 31 ± 6 | 23 ± 5 | <0.001 | 31 ± 6 | 22 ± 5 | <0.001 |

Data are presented as mean ± standard deviation, median (interquartile range), as appropriate.

Abbreviations: DSPN = distal symmetric polyneuropathy; 25(OH)D = 25-hydroxyvitamin D.

**Table S3 Comparison of vitamin D levels stratified by age.**

|  | age < 40 years (n = 35) | | | Age 40–60 years (n = 275) | | | Age >60 years (n = 259) | | |
| --- | --- | --- | --- | --- | --- | --- | --- | --- | --- |
|  | Non-DSPN  (n = 15) | DSPN  (n = 20) | *P* value | Non-DSPN  (n = 75) | DSPN  (n = 200) | *P* value | Non-DSPN  (n = 58) | DSPN  (n = 201) | *P* value |
| 25(OH)D, ng/mL | 27.7 ± 3.3 | 22.1 ± 5.1 | <0.01 | 32 ± 6 | 24 ± 5 | <0.01 | 33 ± 6 | 24 ± 5 | <0.01 |
| 25(OH)D₂, ng/mL | 0.67  (0.53–1.04) | 0.58  (0.49–0.82) | 0.35 | 0.71  (0.58–1.00) | 0.78  (0.58–1.10) | 0.31 | 0.86  (0.60–1.55) | 0.85  (0.59–1.37) | 0.61 |
| 25(OH)D₃, ng/mL | 26.9 ± 3.2 | 21.3 ± 5.1 | <0.01 | 31 ± 6 | 23 ± 5 | <0.01 | 32 ± 6 | 23 ± 5 | <0.01 |

Data are presented as mean ± standard deviation, median (interquartile range), as appropriate.

Abbreviations: DSPN = distal symmetric polyneuropathy; 25(OH)D = 25-hydroxyvitamin D.

**Table S4 Comparison of vitamin D levels after stratification according to BMI.**

|  | BMI <24 ( n = 281) | | | BMI 24–28 ( n = 225) | | | BMI >28(n = 63) | | |
| --- | --- | --- | --- | --- | --- | --- | --- | --- | --- |
|  | Non-DSPN  ( n = 68) | DSPN  (n = 213) | *P* value | Non-DSPN  (n = 60) | DSPN  (n = 165) | *P* value | Non-DSPN  (n = 20) | DSPN  (n = 43) | *P* value |
| 25(OH)D, ng/mL | 32 ± 6 | 24 ± 5 | <0.001 | 33 ± 5 | 24 ± 5 | <0.001 | 30.9 ± 7.5 | 23.3 ± 4.7 | <0.001 |
| 25(OH)D₂, ng/mL | 0.77  (0.56–1.18) | 0.76  (0.54–1.17) | 0.691 | 0.76  (0.61–1.32) | 0.84  (0.61–1.26) | 0.656 | 0.66  (0.59–0.87) | 0.82  (0.67–1.11) | 0.094 |
| 25(OH)D₃, ng/mL | 31 ± 6 | 23 ± 5 | <0.001 | 32 ± 6 | 23 ± 5 | <0.001 | 29.2 ± 5.6 | 22.1 ± 4.6 | <0.001 |

Data are presented as mean ± standard deviation, median (interquartile range), as appropriate.

Abbreviations: DSPN = distal symmetric polyneuropathy; BMI = body mass index; 25(OH)D = 25-hydroxyvitamin D.

**Table S5 Comparison of vitamin D levels stratified by season.**

|  | Winter–Spring (n = 259) | | | Summer–Autumn (n = 310) | | |
| --- | --- | --- | --- | --- | --- | --- |
|  | Non-DSPN ( n = 59) | DSPN ( n = 200) | *P* value | Non-DSPN( n = 89) | DSPN ( n = 221) | *P* value |
| 25(OH)D, ng/mL | 33 ± 7 | 24 ± 6 | <0.001 | 31.6 ± 5.4 | 23.8 ± 4.8 | <0.001 |
| 25(OH)D₂, ng/mL | 0.81 (0.62–1.15) | 0.82 (0.61–1.20) | 0.949 | 0.73 (0.53–1.19) | 0.79 (0.52–1.23) | 0.728 |
| 25(OH)D₃, ng/mL | 32 ± 7 | 23 ± 5 | <0.001 | 30.4 ± 4.9 | 22.8 ± 4.8 | <0.001 |

Data are presented as mean ± standard deviation, median (interquartile range), as appropriate.

Abbreviations: DSPN = distal symmetric polyneuropathy; 25(OH)D = 25-hydroxyvitamin D.

**Table S6 Comparison of MNSI scores across quartiles of serum 25(OH)D levels.**

|  | 25(OH)D group | | | | *P* value |
| --- | --- | --- | --- | --- | --- |
|  | Q1 (8.71–22.10) (n = 142) | Q2 (22.10–25.80) (n = 141) | Q3 (25.80–29.70) (n = 143) | Q4 (29.70–56.60) (n = 143) |  |
| MNSI-Q | 6.00 (5.00–7.00) | 3.00 (1.00–5.00) | 1.00 (0.50–3.00) | 1.00 (0.00–1.00) | <0.001 |
| MNSI-PE | 4.00 (3.00–5.00) | 3.00 (2.00–3.00) | 2.00 (1.00–2.50) | 0.00 (0.00–1.00) | <0.001 |

Data are presented as median (interquartile range).

Abbreviations: MNSI = Michigan Neuropathy Screening Instrument; 25(OH)D = 25-hydroxyvitamin D.

**Table S7 Univariate logistic regression analysis of factors associated with DSPN.**

| Variables | Beta | S.E | Z | *P* | OR (95%CI) |
| --- | --- | --- | --- | --- | --- |
| Age, years | 0.03 | 0.01 | 3.07 | 0.002 | 1.03 (1.01–1.04) |
| BMI, kg/m² | -0.04 | 0.03 | -1.58 | 0.114 | 0.96 (0.90–1.01) |
| SBP, mmHg | 0.01 | 0.01 | 1.86 | 0.063 | 1.01 (1.00–1.02) |
| DBP, mmHg | 0.00 | 0.01 | 0.16 | 0.876 | 1.00 (0.98–1.02) |
| DM duration, years | 0.04 | 0.02 | 2.65 | 0.008 | 1.04 (1.01–1.07) |
| 25(OH)D, ng/mL | -0.32 | 0.03 | -10.67 | <0.001 | 0.73 (0.69–0.77) |
| TG, mmol/L | -0.01 | 0.04 | -0.32 | 0.749 | 0.99 (0.91–1.07) |
| TC, mmol/L | 0.05 | 0.06 | 0.81 | 0.419 | 1.05 (0.93–1.19) |
| HbA1c, % | 0.04 | 0.04 | 1.10 | 0.269 | 1.05 (0.97–1.13) |
| IR | 0.06 | 0.03 | 2.21 | 0.027 | 1.06 (1.01–1.11) |
| Gender |  |  |  |  |  |
| Male |  |  |  |  | 1.00 (Reference) |
| Female | 0.37 | 0.20 | 1.80 | 0.071 | 1.44 (0.97–2.14) |
| Exercise |  |  |  |  |  |
| No |  |  |  |  | 1.00 (Reference) |
| Yes | -0.01 | 0.19 | -0.05 | 0.958 | 0.99 (0.68–1.44) |
| Smoke |  |  |  |  |  |
| No |  |  |  |  | 1.00 (Reference) |
| Yes | -0.03 | 0.22 | -0.16 | 0.875 | 0.97 (0.63–1.49) |

Abbreviations: DSPN = distal symmetric polyneuropathy; BMI = body mass index; SBP = systolic blood pressure; DBP = diastolic blood pressure; DM = diabetes mellitus; 25(OH)D = 25-hydroxyvitamin D; TG = triglycerides; TC = total cholesterol; HbA1c = glycated hemoglobin; IR = insulin resistance; OR = odds ratio; CI = confidence interval.

P values < 0.05 were considered statistically significant.

**Table S8 Multivariable logistic regression analysis of factors associated with DSPN.**

|  | Adj Beta | Adj S.E | Adj Z | Adj OR (95%CI) | Adj *P* |
| --- | --- | --- | --- | --- | --- |
| Age, years | 0.05 | 0.01 | 3.55 | 1.05 (1.02–1.08) | <0.001 |
| BMI, kg/m² | -0.05 | 0.04 | -1.18 | 0.95 (0.87–1.03) | 0.239 |
| SBP, mmHg | -0.01 | 0.01 | -0.76 | 0.99 (0.97–1.01) | 0.445 |
| DBP, mmHg | 0.03 | 0.02 | 1.87 | 1.03 (1.00–1.07) | 0.061 |
| DM duration, years | 0.05 | 0.02 | 2.34 | 1.06 (1.01–1.10) | 0.019 |
| 25(OH)D, ng/mL | -0.37 | 0.03 | -10.68 | 0.69 (0.65–0.74) | <0.001 |
| TG, mmol/L | -0.14 | 0.06 | -2.18 | 0.87 (0.77–0.99) | 0.029 |
| TC, mmol/L | 0.22 | 0.10 | 2.13 | 1.24 (1.02–1.52) | 0.033 |
| HbA1c, % | -0.09 | 0.06 | -1.60 | 0.91 (0.82–1.02) | 0.110 |
| IR | 0.07 | 0.04 | 1.98 | 1.08 (1.01–1.16) | 0.048 |
| Gender |  |  |  |  |  |
| Male |  |  |  | 1.00 (Reference) |  |
| Female | 0.02 | 0.31 | 0.07 | 1.02 (0.56–1.88) | 0.941 |
| Exercise |  |  |  |  |  |
| No |  |  |  | 1.00 (Reference) |  |
| Yes | 0.17 | 0.26 | 0.65 | 1.19 (0.71–1.98) | 0.514 |
| Smoke |  |  |  |  |  |
| Yes |  |  |  | 1.00 (Reference) |  |
| No | 0.09 | 0.33 | 0.28 | 1.09 (0.58–2.07) | 0.783 |

Abbreviations: DSPN = distal symmetric polyneuropathy; BMI = body mass index; SBP = systolic blood pressure; DBP = diastolic blood pressure; DM = diabetes mellitus; 25(OH)D = 25-hydroxyvitamin D; TG = triglycerides; TC = total cholesterol; HbA1c = glycated hemoglobin; IR = insulin resistance; OR = odds ratio; CI = confidence interval.

Adj refers to adjustment for multiple factors.

P values < 0.05 were considered statistically significant.

**Table S9. Threshold effect analysis of serum 25(OH)D levels on MNSI-PE score using the two-piecewise linear regression model.**

| Characteristic | Beta (per SD) | 95% CI | *P* value |
| --- | --- | --- | --- |
| 25(OH)D (< 26.1 ng/mL) | -0.50 | (-0.60 to -0.40) | <0.001 |
| 25(OH)D (≥26.1 ng/mL) | -0.35 | (-0.47 to -0.24) | <0.001 |

Abbreviations: 25(OH)D = 25-hydroxyvitamin D; Beta = regression coefficient; SD = standard deviation; CI = confidence interval.

P values < 0.05 were considered statistically significant.
